# Optical projection tomography as a quantitative tool for analysis of cell morphology and density in 3D hydrogels

**DOI:** 10.1038/s41598-021-85996-8

**Published:** 2021-03-22

**Authors:** Birhanu Belay, Janne T. Koivisto, Jenny Parraga, Olli Koskela, Toni Montonen, Minna Kellomäki, Edite Figueiras, Jari Hyttinen

**Affiliations:** 1 Computational Biophysics and Imaging Group, Faculty of Medicine and Health Technology, Tampere University, Arvo Ylpön Katu 34, 33520 Tampere, Finland; 2 Biomaterials and Tissue Engineering Group, Faculty of Medicine and Health Technology, Tampere University, Tampere, Finland; 3 Heart Group, Faculty of Medicine and Health Technology, Tampere University, Tampere, Finland; 4 Division of Pathology, Department of Laboratory Medicine, Karolinska Institutet, Stockholm, Sweden; 5 HAMK Smart Research Unit, Häme University of Applied Sciences, Hämeenlinna, Finland; 6 Champalimaud Research, Champalimaud Centre for the Unknown, Lisbon, Portugal

**Keywords:** Biophysics, Cell biology, Materials science, Optics and photonics

## Abstract

Assessing cell morphology and function, as well as biomaterial performance in cell cultures, is one of the key challenges in cell biology and tissue engineering (TE) research. In TE, there is an urgent need for methods to image actual three-dimensional (3D) cell cultures and access the living cells. This is difficult using established optical microscopy techniques such as wide-field or confocal microscopy. To address the problem, we have developed a new protocol using Optical Projection Tomography (OPT) to extract quantitative and qualitative measurements from hydrogel cell cultures. Using our tools, we demonstrated the method by analyzing cell response in three different hydrogel formulations in 3D with 1.5 mm diameter samples of: gellan gum (GG), gelatin functionalized gellan gum (gelatin-GG), and Geltrex. We investigated cell morphology, density, distribution, and viability in 3D living cells. Our results showed the usability of the method to quantify the cellular responses to biomaterial environment. We observed that an elongated morphology of cells, thus good material response, in gelatin-GG and Geltrex hydrogels compared with basic GG. Our results show that OPT has a sensitivity to assess in real 3D cultures the differences of cellular responses to the properties of biomaterials supporting the cells.

## Introduction

Tissue engineering (TE) is a fast-growing field that aims to restore the structure and function of diseased or damaged tissue through the use of cells, supportive biomaterials, and biologically active molecules^1^. In TE, various types of biomaterials are used as scaffolds. Among these, hydrogels are becoming increasingly attractive due to their high quantity of water and biocompatibility, while their mechanical and structural properties mimic many soft tissues^1^. Extracellular matrix (ECM)-mimicking hydrogels are thus the key to the progression of cell culture models from flat 2D surfaces to 3D structures that are more representative of human tissues^2^. Hydrogels have recently received attention in drug screening and have been used as 3D culture microenvironments in vitro to predict drug response in vivo^3^. In this paper, we developed a 3D quantitative imaging procedure based on optical projection tomography (OPT) and demonstrate its applicability for the rapid and effective screening of 3D hydrogel cell cultures used for TE applications.

A variety of hydrogels can be produced from synthetic or natural biopolymers or their combinations and can be selectively applied for specific applications based on their physical and biological properties^4^. This creates a need to systematically study their performance as macroscopic scaffolds for cell culturing^5^. During culturing, cell properties can be influenced by a variety of factors, such as interactions with scaffold biomaterials, cell culture times, the density of cells, and cell signaling processes^6^. The microenvironment of cells, such as the surrounding ECM and neighboring cells, define the cell morphology, i.e., size and shape, through adhesive forces and cell-to-cell interactions^7^. Most of our understanding of such biological processes, however, comes from cells cultured on a 2D substrate^8^. Yet, it is well known that there is a significant variation in cell behavior when cells are encapsulated in a 3D environment compared with 2D surface culturing. When cells do not have enough attachment sites, they remain round and inactive^7^. Changes in cell morphology from spherical to a spread or elongated shape are, therefore, a strong indication that the cells prefer their culturing environment^7,9^. Hence, methods to evaluate cell and scaffold properties in 3D in the mesoscopic scale are needed to facilitate the generation of functional tissue in vitro.

To image cells in a 3D cell culture environment, most optical methods can only image samples to a limited depth, which is the major challenge in imaging TE scaffolds^10^. For example, confocal microscopy (CM) has been a useful tool for the high resolution functional imaging of cells^11^. However, CM can only image samples to a depth of up to 300 μm^12^. Although two-photon fluorescence microscopy (TPFM) can provide high-resolution fluorescence images of cell samples at a higher penetration depth (~ 500 µm) and is less phototoxic to live samples when compared with CM, the technique is still limited by its speed and depth of imaging^13^. The development of the selective plane illumination microscopy (SPIM) technique has provided further improvements^13^. SPIM offers better possibilities for the rapid high resolution imaging of transparent samples in the mesoscopic scale with relatively less photobleaching^14^. Similar to CM and TPFM; however, SPIM can only be used to image fluorescence samples.

It is generally recognized that label-free imaging is crucial in cell culturing as it does not perturb normal cell function and enables cells to continue culturing without affecting viability^15^. Two dimensional images from phase contrast microscopy have been used for label-free visualization, the quantification of cell number and morphology in 2D cell culture, and for the evaluation of cells prior to culturing inside a 3D scaffold^16^. Moreover, recent developments in advanced phase imaging enabled the label-free 3D imaging of single cells in thin transparent samples by using optical diffraction tomography reconstruction algorithms^17^. Nevertheless, due to the depth limitation of the methods, they cannot be used for the 3D imaging of cells in a thick 3D biomaterial scaffold^13^. Therefore, there is a need for non-destructive methods that provide valuable and quantitative information to analyze the cells in hydrogels at a higher penetration depth.

Optical Projection Tomography (OPT) is a non-invasive tomographic imaging technique for semi-transparent biomedical samples in the range of 1 mm to 10 mm^12,18^. As in X-ray computed tomography, the specimen is rotated through a series of angular positions, and a two-dimensional projection image is captured at each orientation. In OPT, the sample is suspended in an index-matching liquid to optimize image quality. The standard reconstruction algorithm filtered back projection (FBP) is used to reconstruct the 3D image from the projection images^18^. One of the major advantages of OPT over other techniques is its capability to provide images both in bright-field and fluorescence modes^12^. Moreover, it allows to retrieve the penetration depth in the mesoscopic scale without affecting the viability of cells^12^. Similar to most optical techniques, the main challenge in OPT is the imaging of opaque samples, as this would imply using toxic chemicals for optical clearing^12^. However, transparent hydrogel biomaterials are well suited for OPT and enable mesoscopic imaging. To date, OPT has been applied in developmental biology for studies in small animal embryo anatomy^12^, for 3D visualization of early human brain development^19^, and for in vivo imaging of zebrafish vascular network^20^. Another suitable application of OPT is for characterizing the material properties of hydrogel^18^. Furthermore, OPT has also been applied for the 3D imaging of isolated cell nuclei^21^ and single cells in hydrogel^22^.

In previous work, we have shown that OPT is suitable for characterizing the material properties of hydrogels^18^. However, despite the promising characteristics of OPT, it has not been applied for the assessment of large cell culture samples as a tool to assess biomaterials and their suitability for TE applications.

In this paper, we introduce an OPT imaging and analysis protocol for the quantitative assessment of 3D hydrogels as cell culture environments. To demonstrate the developed protocol and its capability to assess the cellular responses, we applied OPT in comparative studies between three hydrogel formulations: gellan gum (GG) crosslinked ionically with bioamine spermidine^23^, GG crosslinked covalently with gelatin using hydrazone chemistry^2^, and the commercially available Geltrex hydrogel for use as a control biomaterial^24^. With this, we show the usefulness of OPT to assess the cellular responses to the biomaterial environment by qualitative and quantitative analysis of cell morphology, density, distribution, and viability, reflecting how hydrogel formulation influences cell responses. To the best of our knowledge, our paper is the first analysis of the capabilities of OPT in the assessment of biomaterials as cell support.

## Materials and methods

### Materials

Gelatin A from porcine skin, gellan gum (Gelzan CM Gelrite), adipic dihydrazide (ADH), dimethyl sulfoxide (DMSO), ethylene glycol, spermidine trihydrochloride (SPD), 1-ethyl-3-[3 dimethylamino)-propyl]–carbodiimide(EDC), hydroxylamine hydrochloride, N-hydroxybentzotriazole (HOBt), hydrochloric acid, sodium hydroxide, sodium chloride (NaCl), and sodium periodate were obtained from Sigma Aldrich. Geltrex was obtained from Thermo Fisher.

### Chemical modification of biopolymers

To obtain the hydrogels based on covalent interaction, gellan gum (GG) and gelatin were modified to generate hydrazone bonds, according to our previous publication^16^. Briefly, to prepare aldehyde gellan gum (GG-CHO), 500 mg of GG was dissolved in 50 mL water and heated to 60 °C for 1 h. Then, 50 mM sodium periodate solution was added, and the mixture was stirred for 4 h in the dark. Ethylene glycol was then added to stop the reaction. The polymer was dialyzed against water for 4 days, followed by freeze-drying.

To prepare hydrazide gelatin (gelatin-ADH), 300 mg of gelatin were dissolved in 100 mL water. To this solution was added 3.92 g of ADH. The pH of the reaction mixture was adjusted to 6.8 with 0.1 M NaOH and 0.1 M HCl. Then, 576 mg of EDC and 405 mg HOBt were dissolved in 3 mL DMSO/water (1.5:1 v/v). The EDC/HOBt mixture was then added to the reaction mixture drop by drop keeping the pH at 6.8. In addition, the pH was adjusted and kept at 6.8 for 4 h. The reaction was then kept ongoing for a further 20 h without pH adjustment. After this period, the pH was adjusted to 7 and gelatin-ADH was exhaustively dialyzed against water for 2 days. NaCl was added to produce a 5% (w/v) solution and the product was precipitated in cold ethanol. Then, the product was dissolved in water and dialyzed against water for 2 days, followed by freeze-drying.

### Hydrogel formulation

For this study, we prepared hydrogels based on two crosslinking strategies. First, GG hydrogels based on ionic crosslinking were obtained by the interaction of native GG (5 mg/mL) with the bioamine spermidine (SPD) (0.5 mg/mL). SPD acts as a polycation, generating ionically crosslinked GG hydrogels. The solutions were prepared in sucrose 10% (w/w) and sterile filtered using a Whatman FP 30/0.2 CA-S sterile filter (Thermo Fisher Scientific, USA) at 37 °C. SPD was mixed with GG at a volume ratio of 4:25 and cast into a suitable mold.

Second, we obtained hydrogels based on chemical crosslinking by the generation of hydrazone bonds. GG-CHO and gelatin-ADH were dissolved in 10% sucrose to make 20 mg/mL and 40 mg/mL solutions, respectively. To prepare gelatin-ADH/GG-CHO hydrogels (gelatin-GG), the solutions were mixed at 1:1 volume ratio by pipetting. Gelatin-ADH and GG-CHO solutions were sterile filtered according to our previous publication^2^. During the hydrogel preparation, all the solutions were kept at 37 °C to avoid thermal shock to the cells. In addition, we used Geltrex hydrogel as a control, which was prepared according to the manufacturer’s instructions utilizing thermal gelation when heated to 37 °C.

### Cell culture protocol

The commercial human lung fibroblast cell line WI-38 (Culture Collections, Public Health England, United Kingdom) was expanded and cultured in Nunc T75 culture flasks (Thermo Fisher Scientific, USA) with Dulbecco’s Modified Eagle Medium/Ham’s Nutrient Mixture F-12 (DMEM/F-12 1:1; Thermo Fisher Scientific, USA) supplemented with 10% fetal bovine serum (South American origin, Biosera, Finland) and 0.5% Penicillin/Streptomycin 100 U/mL (P/S; Thermo Fisher Scientific, USA). For the hydrogel cell culture, cells were detached with trypsin (Lonza), counted, and encapsulated in the hydrogels (GG, gelatin-GG or Geltrex) with a cell density of between 300,000 cells/mL and 1,000,000 cells/mL. The cells were cultured in the encapsulated condition in the different hydrogels for 7 days.

### Sample preparation for OPT imaging

For OPT imaging, the hydrogel cell cultures were prepared in the custom-made polydimethylsiloxane (PDMS) platforms. The PDMS was fabricated from Sylgard 184 base polymer and curing agent (10:1, w/w, Sylgard 184, Dow Corning, USA). Sylgard 184 was acquired from Ellsworth Adhesives AB (Sweden). Hydrogel cell cultures with fibroblasts were prepared inside a fluorinated ethylene propylene (FEP, Adtech Polymer Engineering, UK) tube in the custom-made 3D printed PDMS platform. The cell medium was added on the top of the cell-hydrogel.

For bright-field OPT imaging, the cell culture medium was removed from the FEP tube, and cell-laden hydrogels were taken by pumping a portion of the sample into an FEP tube. In all our OPT imaging, we used an FEP tube with an inside diameter of 1.5 mm. The refractive index of the FEP tube (n ~ 1.33) is close to water, so the light scattering is minimal, which is essential for OPT imaging.

To visualize and measure cell viability in the 3D hydrogel, the cells were stained using a Live/Dead viability assay (Thermo Fisher Scientific, USA). The staining solution, containing 0.1 µM ethidium-1 (stains dead cells) and 0.4–0.6 µM Calcein AM (stains live cells), was prepared in phosphate buffered saline (PBS, Lonza), following the recommendations of the manufacturer. The fluorescence staining method was optimized for the OPT imaging of cells in a 3D hydrogel. To enhance the stain diffusion throughout the sample, a puncture hole was made in the middle of the cell-laden hydrogel, and the stain solution was added through the hole. Then, the sample was incubated for 1 h at + 37 °C. The stained samples were then taken by pumping a portion of the sample into a 1.5 mm inside diameter FEP tube and transferred for fluorescence OPT imaging, similar to what is made in bright-field OPT.

### Standard phase contrast microscopy imaging

To visualize the morphology and size of fibroblasts before culturing in hydrogels, the cells were first detached from the flask dish with trypsin and imaged using phase contrast microscopy (Zeiss digital microscope AxioCam MRc5 microscopy camera (Carl Zeiss MicroImaging GmbH, Germany)) with a 5x, NA 0.15 objective.

### Bright-field OPT image acquisition

**Figure 1:**
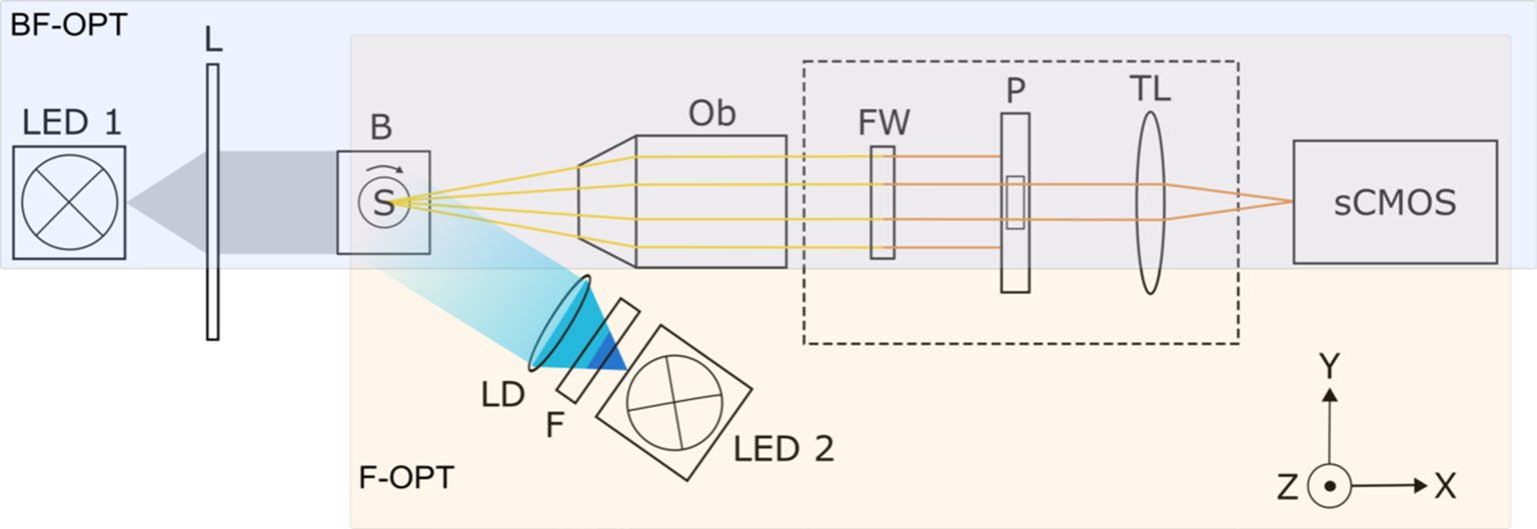
Schematic diagram of the OPT setup with both transmission (BF-OPT) and emission (F-OPT) modes represented: samples inserted in FEP tubes are rotated by the rotation stage (S) inside an index-matching water bath (B). A white light (LED 1) and a telecentric lens (L) are used for bright-field imaging in OPT. Fluorescence illumination provided by a LED (LED 2), a bandpass filter (F), and a collimated lens with a diffuser (LD) in fluorescence OPT mode. The detection system comprises an objective lens (Ob), a rotating filter wheel (FW) used as a band-pass filter (only for fluorescence imaging), a pinhole (P), a tube lens (TL), and a sCMOS camera.

In this study, an in-house built OPT system was optimized for the imaging of cells in 3D hydrogels. A schematic diagram of the system can be seen in Fig. 1. A more detailed description of the system is given in^18^.Since then, the system has been updated with light sources and filters better suited for Live/Dead cell nuclei (DAPI)-staining. The system works in both bright-field and fluorescence modes, shown as BF-OPT (with blue background color) and F-OPT (with yellow background color), respectively, in Fig. 1. Bright-field OPT was used for the visualization and quantitative morphological analysis of fibroblasts in 3D hydrogels. The samples were prepared in an FEP tube and submerged inside a cuvette filled with water for imaging. A white light emitting diode (LED) source and a telecentric backlight illuminator (Edmund, USA) was used to illuminate the sample (LED 1 in Fig. 1). The transmitted light was detected by a 5 × infinity-corrected objective (Ob, Edmund, USA) with a numerical aperture (NA) of 0.14 and imaged with an sCMOS camera (ORCA-Flash 4.0, Hamamatsu, Japan). The rotational center of the sample was manually aligned using x–y-stage (Standa, Lithuania). The sample was rotated 360 degrees while a total of 400 projection images were captured at 0.9-degree intervals. It took upto 10 min to image full 400 projection images.

### Fluorescence OPT image acquisition

For fluorescence imaging of fibroblasts in the 3D hydrogel, fluorescence OPT with epi-illumination was used. A LED source (M470L2, Thorlabs) with an excitation wavelength of 470/30 nm and an emission bandpass filter of λ = 520 ± 36 nm was used for live cell imaging. In addition, a LED source (M530L3, Thorlabs) with an excitation wavelength of 530/33 nm and an emission bandpass filter of λ = 623 ± 24 nm was used for dead cell imaging. All projection images were acquired with a 5 × objective lens and 3 s exposure, yielding a total 40–50 min imaging time, for combined Live/Dead fluorescence imaging. The LED sources were supplied with a constant current of 500 mA.

### Bright-field OPT image processing and analysis

**Figure 2:**
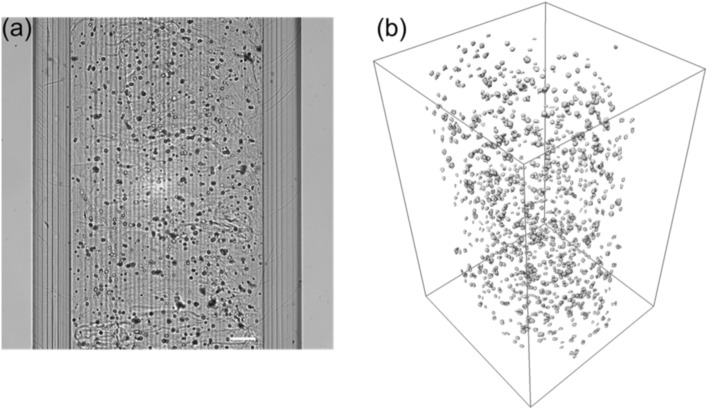
Bright-field OPT imaging of fibroblasts in a 3D GG hydrogel. (**a**) Raw bright-field projection image, and (**b**) 3D visualization of segmented cells. The scale bar indicate 200 µm.

Image processing and reconstruction were applied to quantitatively evaluate the morphology and number of the cells in a 3D hydrogel. The bright-field projection images (Fig. 2a) were processed with a homomorphic filter to normalize image brightness across the image stack, as described previously^18^. Volumetric 3D reconstructions were computed in MATLAB (MathWorks) using a filtered back-projection algorithm (FBP) with manual center-of-rotation correction^18^. Then, the reconstructed 3D image stacks were inverted for better visualization, and the cells were segmented from the background using Avizo (Thermo Fisher Scientific, Waltham, MA, USA) ‘Label Field’ segmentation editor. In the segmentation editor, we first used intensity thresholding by manually adjusting the minimum and maximum image intensity, and then we applied mathematical morphological dilation and erosion operations in 3D. To evaluate the segmentation accuracy, the resulting segmented 3D images of cells were compared with the direct visualization of cells in 3D. Small artifacts arising from hydrogel texture, acquisition, and reconstruction were removed using a small particle removal filter in Avizo. Also using Avizo, volume was rendered to visualize cells in 3D (Fig. 2b).

After 3D segmentation of cells provided the 3D cell locations and shapes, we used the label analysis module in Avizo to quantify cell shape parameters using the segmented 3D image stack. The parameters of elongation and flatness were determined to quantitate the variation in morphology of fibroblasts cultured in different hydrogel formulations.

In our analysis, elongation is defined as the ratio of the largest to the medium radii of the cell

1 $${\text{Elongation}} = {\text{R}}_{{{\text{largest}}}} /{\text{R}}_{{{\text{medium}}}}$$

where elongated cells have a value much greater than one. Flatness is defined as the ratio of the medium to the smallest radii of the cell.

2 $${\text{Flatness}} = {\text{R}}_{{{\text{medium}}}} /{\text{R}}_{{{\text{smallest}}}}$$

and flat cells have a value much greater than one.

### Fluorescence OPT image processing and analysis

The images were brightness-adjusted for better contrast, and a median filter was used to remove random photon noises cause by the microscope and detector. To create a composite image and a video of live and dead cells, a projection image of dead cells was aligned to a corresponding projection image of live cells using Fiji software.

### Statistical analysis

The measured quantitative data were analyzed using Stata Statistical Software (StataCorp.2017). Shapiro–Wilk test was used to evaluate the normality of the datasets. For parametric tests, the groups were compared using analysis of variance (One-way ANOVA). Pairwise comparison between the groups was determined with Bonferroni multiple comparisons test. For non-parametric tests, the groups were compared using non-parametric Kruskal–Wallis rank test with Dunn’s pairwise comparison test. For mean comparison between two groups, T-test was applied. Statistical significance was determined to be *P* < 0.05.

## Results

### Bright-field OPT imaging for comparative analysis of hydrogel formulations

**Figure 3:**
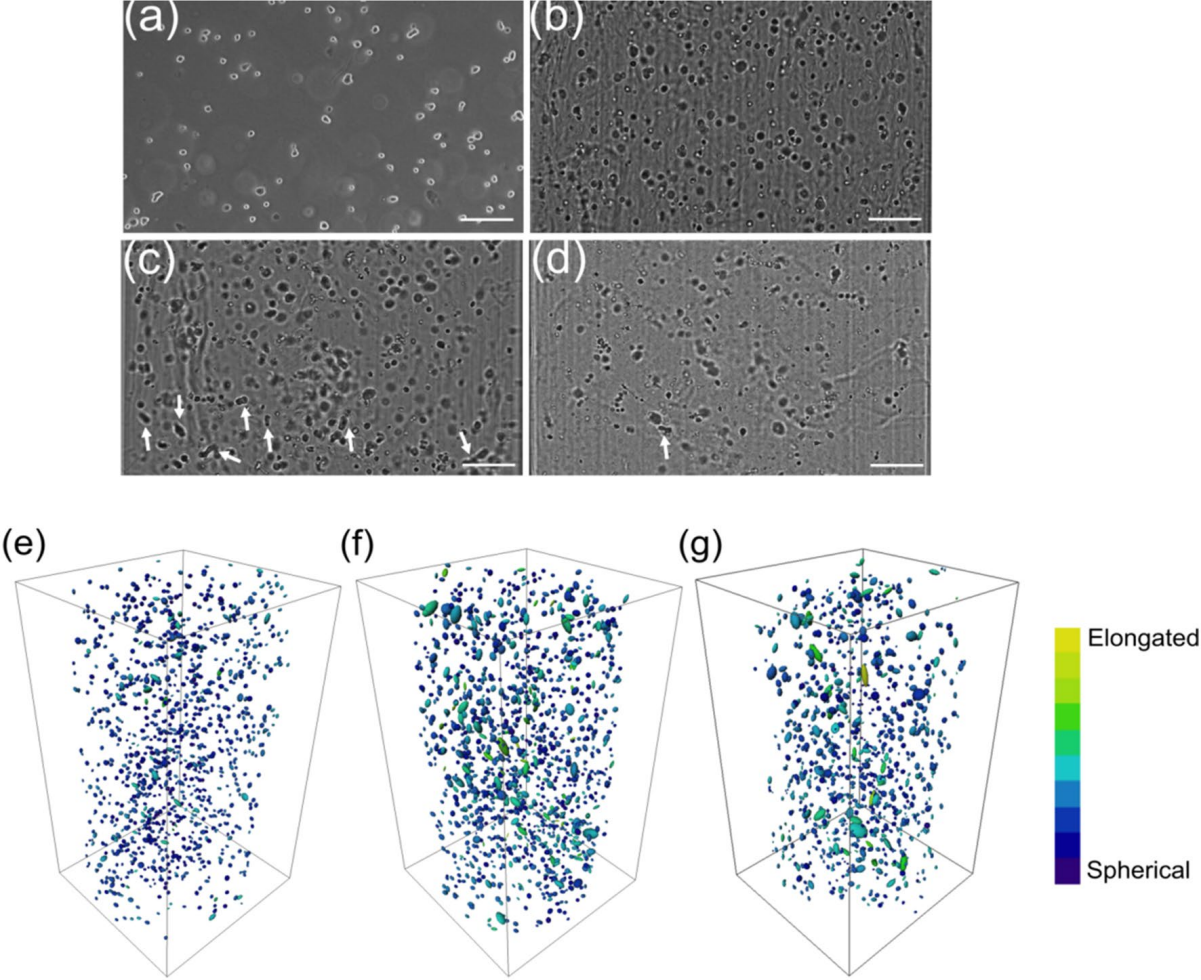
(**a**) 2D phase contrast image of human lung fibroblast (WI-38) cells in a culture flask acquired using a 5 × objective; representative OPT bright-field projection images of the same cells cultured for 7 days in (**b**) GG, (**c**) gelatin-GG, and (**d**) Geltrex hydrogel; (**e**–**g**) visualization of 3D reconstructed images of cells in (**b**–**d**)). The arrows indicate large size and elongated cells. The color scale was assigned with darker blue indicating cell sphericity to lighter green and yellow indicating cell elongation. The scale bar in the images represents 200 µm.

Human lung fibroblast cells imaged using phase contrast microscopy and bright-field OPT are shown in Fig. 3. In the phase contrast image shown in Fig. 3a, we can see the rounded shape and small size of the fibroblasts in suspension before being encapsulated in the 3D hydrogel. A similar size and morphology of cells can be seen in the GG hydrogel. 2D projection images of the cells in each GG, gelatin-GG, and Geltrex hydrogel are shown in Fig. 3b–d, respectively, where we can see the spatial distribution of the cells. Visual investigation revealed that more elongated cells are found in the protein functionalized gelatin-GG and Geltrex hydrogels (see arrows in Fig. 3c,d) when compared with the cells cultured in the GG hydrogel.

In Fig. 3e–g, we present the 3D reconstructed images of cells in different hydrogels. As can be seen from the images, the cells are uniformly distributed along all directions in 3D in all hydrogel formulations. Even though the number of seeded cells were the same, a higher density of cells in gelatin-GG (Fig. 3f) was observed when compared with the GG and Geltrex hydrogels. The most noticeable difference is observed in the morphology of cells highlighted by the false colors scale, from darker blue indicating sphericity to yellow indicating elongation. Figure 3e shows small cells, similar to Fig. 3b. In contrast, the cells cultured in gelatin-GG (Fig. 3f) and Geltrex hydrogel (Fig. 3g) are larger in size with more elongated morphology. The overall distribution of fibroblasts cultured in the GG, gelatin-GG, and Geltrex hydrogels are similar (Supplementary Video 1).

**Figure 4:**
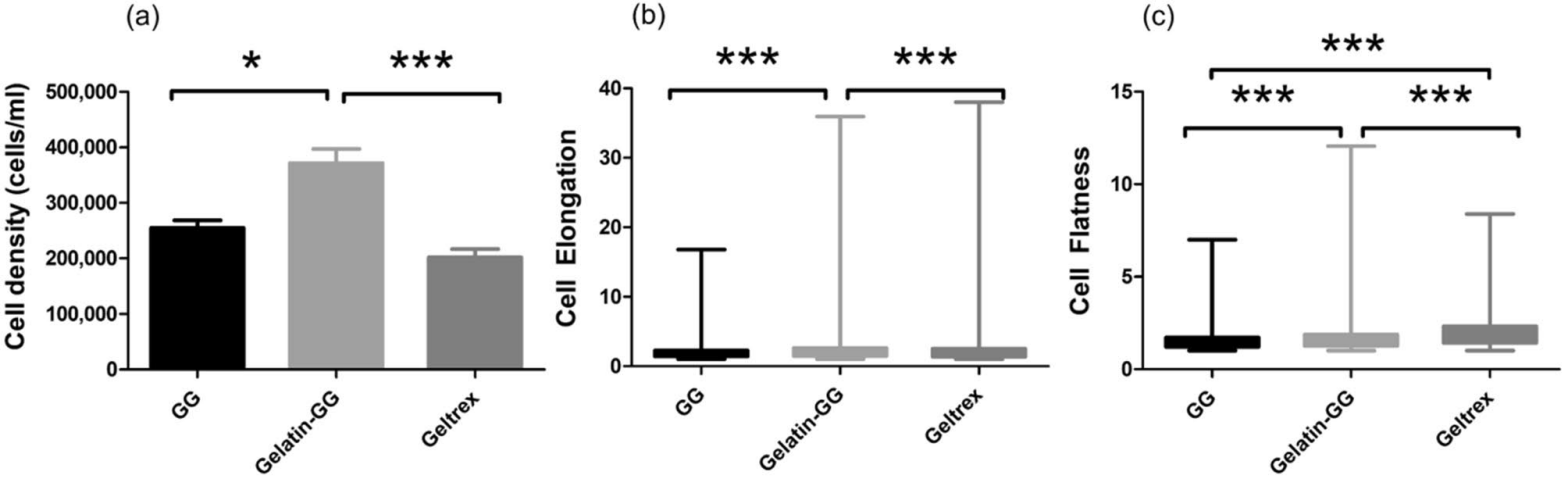
Quantative results of cell density and elongation for human lung fibroblasts cultured for 7 days in three different hydrogel formulations (GG, Gelatin-GG, and Geltrex) plotted using bar chart and a Box-Whiskers diagram; (**a**) cell density, (**b**) cell elongation, and (**c**) cell flatness. One-way ANOVA with Bonferroni multiple comparisons test was used to compare density of cells in different hydrogel formulations. Cell elongation and flatness were analyzed using non-parameterric Kruskal–Wallis rank test with Dunn’s pairwise comparison test. n = 3, (*P < 0.05; **P < 0.01; ***P < 0.001).

The quantitative results of cell density and shape for fibroblasts cultured for 7 days in three different hydrogel formulations are shown in Fig. 4. The quantified mean cell density in gelatin-GG (mean = 371,347 cells/mL, SD = 25,435) is significantly higher than in Geltrex (mean = 201,773 cells/mL, SD = 15,142) and GG (mean = 254,751 cells/mL, SD = 13,883) (see Fig. 4a) showing better cell proliferation in the gelatin-GG hydrogel. The 3D cell shape parameters (elongation and flatness) were quantified to show how the fibroblasts interact with their microenvironment in 3D. Figure 4b,c shows the cell elongation described by Eqs. (1) and (2). The fibroblast were highly elongated with a significantly higher average elongation value in gelatin-GG (median = 1.83, Q_1_ = 0.66 Q_2_ = 3) when compared with the cells in GG (median = 1.75, Q_1_ = 0.92 Q_2_ = 2.58) and Geltrex (median = 1.67, Q_1_ = 0.51 Q_2_ = 2.83) hydrogels. Similarly, Fig. 4c shows that fibroblast were flatter with higher flatness values in Geltrex (median = 1.8, Q1 = 0.92 Q3 = 2.68) and gelatin-GG (median = 1.48, Q1 = 0.87 Q3 = 2.09) when compared with cells in the GG (median = 1.4, Q1 = 0.9 Q3 = 1.9).

### Bright-field OPT for analysis of cell distribution and density in GG hydrogel

**Figure 5:**
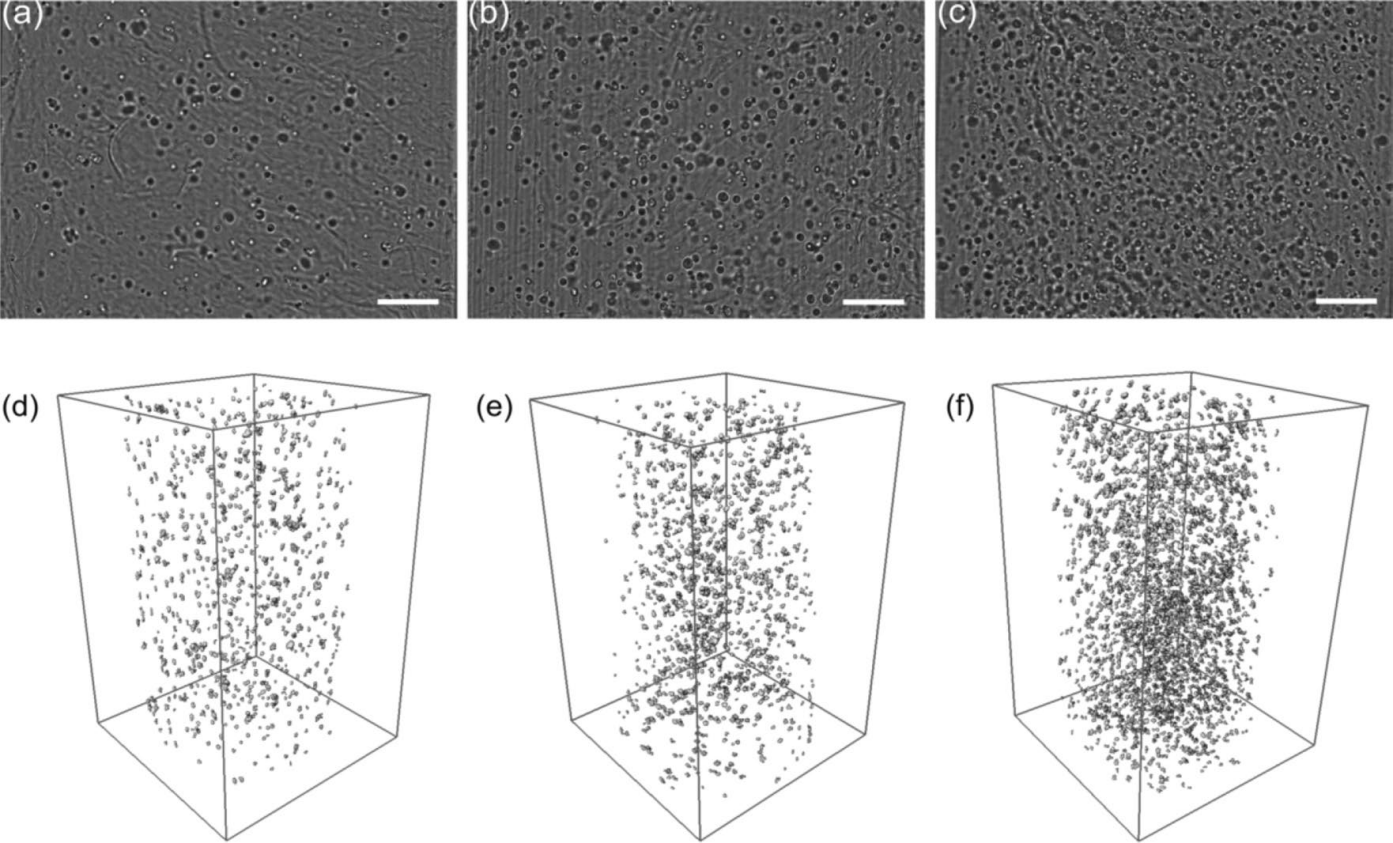
Bright-field OPT projection images of human lung fibroblast (WI-38) cells in 3D GG hydrogel after 7 days culturing with initial cell seeding density of (**a**) 300,000 cells/mL, (**b**) 500,000 cells/mL, and (**c**) 1,000,000 cells/mL; (**d**–**f**) 3D reconstructed images of cells in (**a**–**c**)), respectively. The scale bar in the images represents 200 µm.

To demonstrate the analysis of cell density and distribution with label-free bright-field OPT, we imaged the GG hydrogel with a different initial number of cells: 300,000 cells/mL, 500,000 cells/mL, and 1,000,000 cells/mL. Bright-field OPT projection images of fibroblasts in 3D hydrogel with different initial seeded cell densities and respective reconstructions can be seen in Fig. 5 and Supplementary Video 2. For the cell densities of 300,000 cells/mL and 500,000 cells/mL, the cells were sparsely spread throughout the hydrogel, as shown in Fig. 5a,b,d,e. However, with a larger cell density of 1,000,000 cells/mL (Fig. 5c,f), the cells were highly dense and evenly distributed throughout the hydrogels.

The relationship between the quantified mean cell density and the initial cell density is shown in Table 1. The quantified mean cell density was much smaller than the initial cell density for each sample and a significant statistical difference was also observed. This is important to take into account in study design.

**Table 1:** The measured initial density of cells and the quantified cell density after culturing of the cells for 7 days in GG hydrogel.

Initial seeded cell density (number of cells/mL)	300,000***	500,000*	1,000,000**
Quantified cell density (average number of cells/mL)	169,007	307,518	563,262
Fraction of quantified density with respect to the initial	56%	62%	56%

T-test was applied to compare between the initial and quantified cell density. n = 3, (*P < 0.05; **P < 0.01; ***P < 0.001).

### Fluorescence OPT for assessment of cell viability, distribution, and morphology

**Figure 6:**
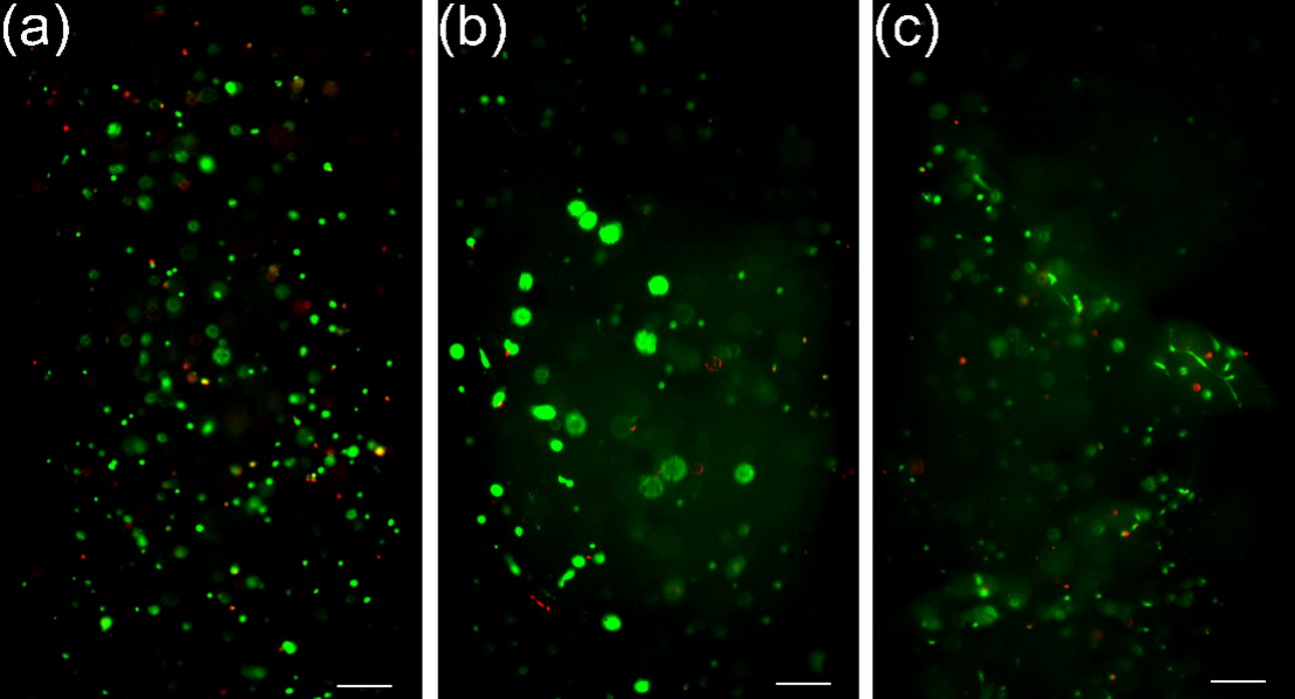
Fluorescence OPT projection images of live (green) and dead (red) fibroblasts cultured for 7 days in 3D (**a**) GG, (**b**) gelatin-GG, and (**c**) Geltrex hydrogels. The scale bar in the images represents 200 µm.

Here, we compared the viability and morphology of fibroblasts cultured for 7 days in the three different hydrogel formulations. Example fluorescence OPT live/dead composite projection images of fibroblasts in GG, gelatin-GG, and Geltrex hydrogels can be seen in Fig. 6a–c, respectively. The intense green colors are associated with live cells and the intense red colors are associated with dead cells. The projection images revealed high cell viability in all three hydrogel formulations over the 7-day culture period. However, as shown in Fig. 6b,c, the cells in gelatin-GG and Geltrex hydrogels are larger in size and more elongated in morphology and are similar to the bright-field OPT images previously shown in Fig. 3. In contrast, the cells cultured in GG remained rounded and small in size (See Fig. 6a). Moreover, cells that are connected to each other can be seen in the Geltrex hydrogel, as shown in Fig. 6c. We observed that the cells were not uniformly distributed throughout the gelatin-GG and Geltrex hydrogels because a region with no cells can be seen in the top right corner of the samples (Fig. 6b,c). OPT live/dead projection videos provided a more complete view of the shape, distribution, and viability of cells throughout the volume of hydrogel (Supplementary Video 3).

## Discussion

The ability to quantitatively evaluate macroscopic hydrogel for 3D cell culture and TE applications is highly required. In this work, we used OPT to analyze fibroblast 3D cell morphology in three different hydrogel formulations in order to show how OPT can be used to assess the biomaterial effects on cells. Based on our results, we found that fibroblasts in gelatin-GG and Geltrex had highly spread morphology throughout the scaffold when compared with the small and round cells in the GG hydrogel. This was observed in both bright-field and fluorescence OPT results. The result highlights the suitability of these hydrogels for TE applications, which has also been shown with other methods^2,7,23^. This highlights the capability of the proposed methods to assess hydrogel biomaterials. Between gelatin-GG and Geltrex, the difference was quantified in cell density and elongation after 7 days of cell culturing, with more elongated and also a higher number of cells in gelatin-GG. In this case, the cells proliferated because gelatin provides the key peptides, i.e., arginine-glycine-aspartic acid (RGD), and matrix metalloproteinase (MMP) degradable features^2^. However, the traditional live/dead staining– even performed in 3D, was not able to show these less profound effects as no visible difference in cell viability was observed among the hydrogel formulations.

The ability to quantitate 3D cell morphology and density is the key to achieving statistically proven results on the effect of hydrogel formulation on cell morphology and proliferation. In this study, we conducted a quantitative density analysis from the bright-field OPT 3D reconstructions of fibroblasts in GG hydrogel samples. The quantified number of cells after seven days of culturing was from 56 to 62% of the initially seeded cell density. This could be due to the death of cells, either during sample preparation or during culture. In cell death, the loss of cell membrane integrity yields a decrease in the optical contrast of dead cells. Hence, the dead cells were not detected from bright-field OPT images. This was further verified using cell viability analysis using OPT in fluorescent mode (Figs. S1, S2), where the samples were stained with LIVE/DEAD assay. The detected fibroblast viability in the GG hydrogel after seven days of culturing was 64.3%. This result agrees with the number of cells detected in the bright-field OPT reconstruction. Furthermore, another source for the low quantified cell density can be addressed to human error, mainly cell counting during sample preparation using a Bürker counting chamber. Thus, quantifying the cell density can actually be used a method to verify sample preparation accuracy.

As shown in the fluorescence images and videos (Fig. 6b,c and Supplementary Video 3) of gelatin-GG and Geltrex, there is a region in the top right corner with no cells. This variation in the spatial distribution of cells is an example that shows that large 3D volume analysis of cell viability is essential for the proper evaluation of the biocompatibility of hydrogels. The homogenous distribution of cells and their density in a 3D biomaterial scaffold is vital to enhance functional tissue generation in vitro by improving cell-to-cell and cell-ECM interactions^7^. For example, the seeding of cardiac cells with high density homogeneously throughout the scaffold could help to maintain the high metabolic activity of cells for weeks in vitro in 3D cell culture^25^. Then again, high cell density can cause detrimental effects via hypoxia to sensitive cells^10^. Hence, controlling and monitoring the cell density during seeding and culturing needs to be optimized according to the specific application. To address these issues, as we have shown here, OPT provides a suitable tool for fast mesoscopic volume imaging and quantitative assessment.

Despite our promising results with all tested hydrogels, the OPT system still faces challenges in the imaging of more opaque samples due to light attenuation restricting light penetration through turbid media. For example, the high spreading of cells in 3D can cause an increase in the sample opacity and cell to cell contact. This could affect single-cell identification and quantification of cell morphology parameters. Furthermore, the limited depth of focus (DOF) of an objective lens poses challenges for 3D reconstructions. Since only cells close to the focal plane are captured accurately in the projections, the radial quality of 3D reconstructions decreases. Several approaches to overcome the limitation in the depth of focus have been proposed either through focal adjustment^26^ or by different mathematical approaches^27,28^, which seem a promising direction for the improvement of the reconstruction in cell and small animal model imaging, especially OPT in fluorescence mode. In addition, it is possible to increase the focal content of the images through multifocal acquisitions using a high NA lens^26^. To overcome the limited depth-of-focus caused problems, in this study, we used a low numerical aperture (NA = 0.14) lens to obtain a suitable DOF for imaging cells in a large sample. For fluorescence OPT, the physically relevant modeling of light propagation is suggested to improve the method since the detection of emission light cannot be accurately modeled using transmission Radon transform^29^. Nonetheless, the fluorescence reconstructions obtained in this work using a basic Radon transfer method were able to quantify the number of live and dead cells from 3D volumes.

In addition, when culturing a high density of cells (> 1 million cells per mL) we observed the formation of clusters of cells and cell to cell contact or ECM produced by cells and this pose issues in the transparency of the sample and resolving single cell morphology or analyzing cell density. As we show here (Fig. S3a), in such case of cell clusters, fluorescence OPT with DAPI staining can provide tools for the visualization of fluorescence projection images showing cell nuclei and their distribution also in cell clusters in GG hydrogel. This result demonstrates that the functional information from OPT in fluorescence mode complements the structural information provided by OPT in bright-field mode. Likewise, out previously published results with analyzing osteogenic differentiation show that ECM and even mineralization produced by cells can be imaged with OPT to a certain^30^. However, there is still a need to improve the methods to allow the proper diffusion of stain molecules in large volume scaffolds, leaving room for improvements in 3D cell staining methods. Furthermore, the continuous development of different types of fluorescent dyes and staining methods will in future increase the capabilities of fluorescence OPT with a wide range of biological applications including gene and protein expression, molecular interactions in cells, and cell-to-cell interactions in 3D.

To summarize, the results in this work have demonstrated that OPT can be used for any label-free cell morphology and density analysis as well as for fluorescence tagged studies in 3D transparent biomaterial scaffolds. The results highlight the ability of the OPT to compare the cellular responses within various hydrogel microenvironments in large volume samples, and thus evaluate hydrogels as biomaterials. In conclusion, OPT offers an excellent and relatively cheap platform for the 3D quantitative imaging of cell-hydrogel samples taking only a few minutes at a penetration depth of up to 1 cm. In the future, we plan to implement an incubator, with controlled temperature and CO_2_ concentration, in the OPT setup for long term imaging of cell culture, and thus follow tissue formation in 3D. We are also aiming to extend the depth of field of imaging using multifocal imaging to image large depth for improved 3D reconstruction. This would also allow us to use high magnification lens for better visualization of cell morphology.

## Supplementary Information

Supplementary Information 1.

Supplementary Information 2.

Supplementary Information 3.

Supplementary Information 4.

Supplementary Information 5.

Supplementary Information 6.

Supplementary Information 7.

Supplementary Information 8.

Supplementary Information 9.

Supplementary Information 10.

Supplementary Information 11.

Supplementary Information 12.

Supplementary Information 13.
