# Supplementary material for: Optical projection tomography as a quantitative tool for analysis of cell morphology and density in 3D hydrogels
Source: Sci Rep. 2021 Mar 22;11:6538. doi: 10.1038/s41598-021-85996-8 (PMC7985381; doi:10.1038/s41598-021-85996-8)
Supplement: Supplementary file 1 — Supplementary Information 1. [file 41598_2021_85996_MOESM1_ESM.docx]

**Supplementary Figures**

**Optical projection tomography as a quantitative tool for analysis of cell morphology and density in 3D hydrogels**

Birhanu Belay^1^*, Janne T. Koivisto^2,3,4^, Jenny Parraga^2^, Olli Koskela^1,5^, Toni Montonen^1^, Minna Kellomäki^2^, Edite Figueiras^6^, Jari Hyttinen^1^

^1^Computational Biophysics and Imaging Group, Faculty of Medicine and Health Technology, Tampere University, Arvo Ylpön katu 34, Tampere, Finland

^2^Biomaterials and Tissue Engineering Group, Faculty of Medicine and Health Technology, Tampere University, Tampere, Finland.

^3^Heart Group, Faculty of Medicine and Health Technology, Tampere University, Tampere, Finland

^4^Division of Pathology, Department of Laboratory Medicine, Karolinska Institutet, Stockholm, Sweden

^5^HAMK Smart Research Unit, Häme University of Applied Sciences, Hämeenlinna, Finland

^6^Champalimaud Research, Champalimaud Centre for the Unknown, Lisbon, Portugal

Corresponding author

Birhanu Belay

Arvo Ylpön katu 34, 33520, Tampere, Finland

[birhanu.belay@tuni.fi](about:blank),

+358402157322


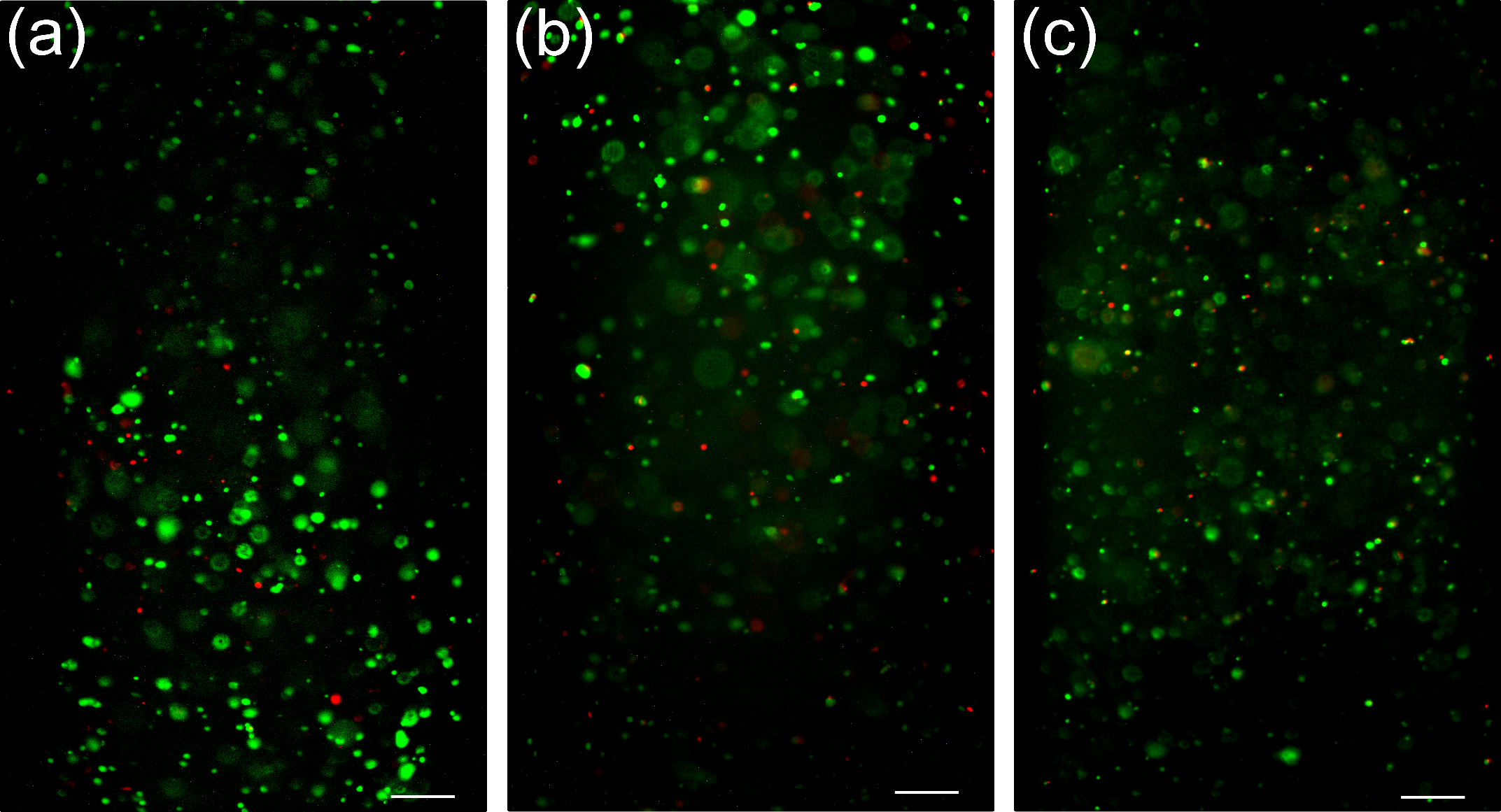


Fig.S1. Fluorescence OPT projection images of live (green) and dead (red) fibroblast cells cultured in GG hydrogel in vitro for (a) 1 day, (b) 3 days, and (c) 7 days. Most of the fibroblast cells remained viable for the duration of the cell-culture time and were uniformly distributed throughout the biomaterial scaffold. The scale bar in the images represents 200 µm.


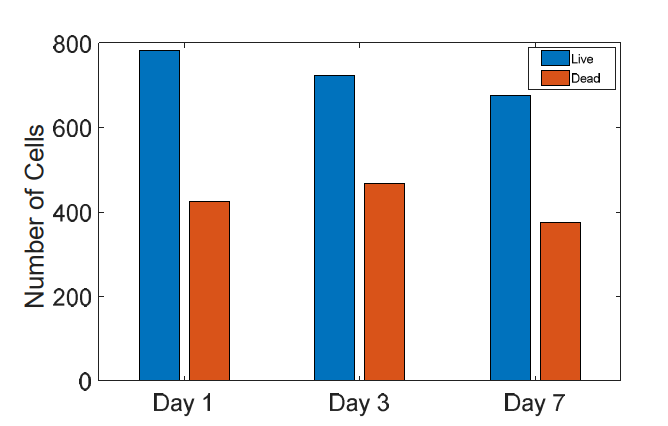


Fig.S2. Quantified number of live and dead cells in GG hydrogel for images shown in Fig.S1. To quantify the number of live and dead cells, the 3D images of live/dead cells were reconstructed from the projection images using the FBP algorithm. Manual intensity thresholding was applied to segment cells from background. The Avizo label analysis module was used for automatic quantification of live and dead cells from images of the segmented cells. The cell viability was determined as the ratio of live cells and the total number of cells. The calculated percentage of viable cells after 1, 3, and 7days of culturing was 64.8, 60.8, and 64.3, respectively. There was therefore no visible difference in cell viability with respect to culture time.


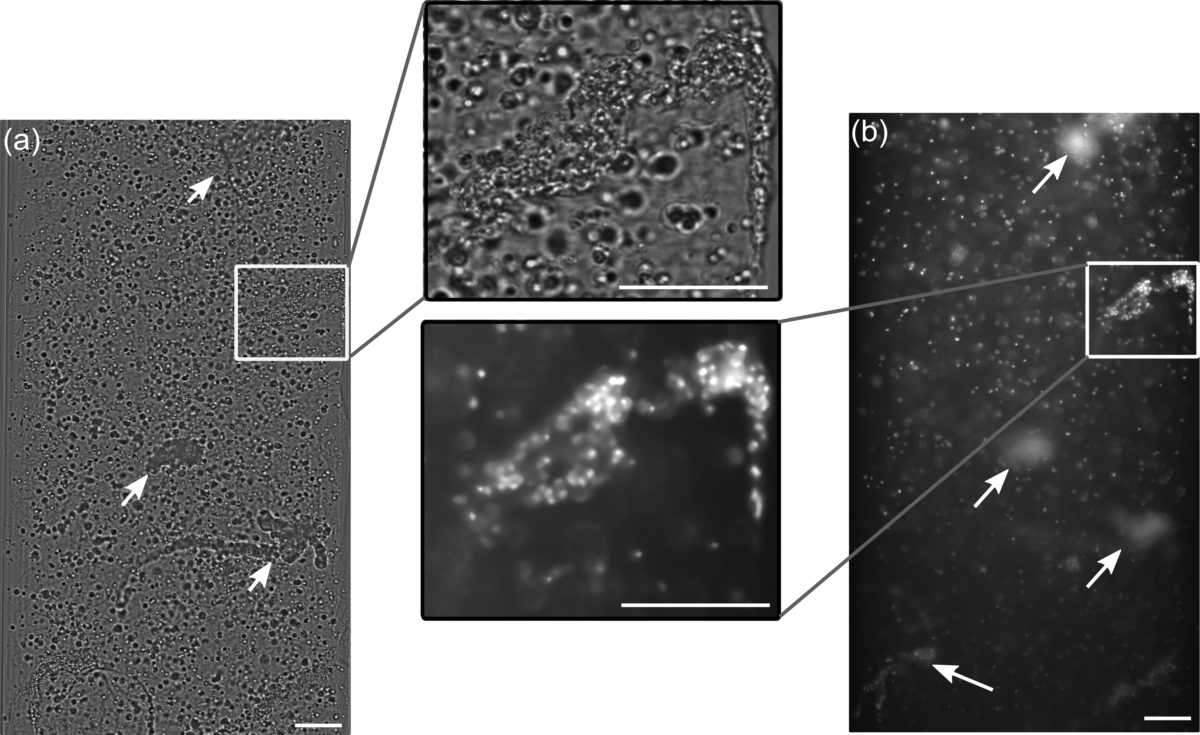


Fig.S3. Representative OPT (a) bright field projection image of cells in GG hydrogel, and (b) fluorescence projection images of cell nuclei. The samples were stained with4,6-diamidino-2-phenylindole (DAPI). During the staining procedure, the cell samples were fixed with 4% paraformaldehyde for 30 minutes. After fixation, the samples were stained with 0.5 μg/mL DAPI aqueous solution for 45 minutes at room temperature. Washing with PBS three times was done between each step. The DAPI stained samples were stored in PBS at 4 °C and light protected before imaging, and imaged in fluorescence mode, a LED source (M65L2, Thorlabs) with an excitation wavelength of 365/7.5 nm and an emission bandpass filter of λ= 460±50 nm was used). The arrows indicate out-of-focus cell clusters. As shown in the box in (a), cells form large clusters, and it can be difficult to identify individual cells from the bright field OPT image. However, as shown in (b), the cell nuclei are visible and resolved in the fluorescence OPT image. Moreover, the out-of-focus cell clusters are seen in both the bright field and fluorescence projection images (see arrows). A video showing single cells and a cluster of cells from various angles can be seen in Supplementary Video 4. The scale bar in the images represents 200 µm.
