# Supplementary material for: Optical projection tomography as a quantitative tool for analysis of cell morphology and density in 3D hydrogels
Source: Sci Rep. 2021 Mar 22;11:6538. doi: 10.1038/s41598-021-85996-8 (PMC7985381; doi:10.1038/s41598-021-85996-8)
Supplement: Supplementary file 2 — Supplementary Information 2. [file 41598_2021_85996_MOESM2_ESM.docx]

**Supplementary Video Material**

**Optical projection tomography as a quantitative tool for analysis of cell morphology and density in 3D hydrogels**

Birhanu Belay^1^*, Janne T. Koivisto^2,3,4^, Jenny Parraga^2^, Olli Koskela^1,5^, Toni Montonen^1^, Minna Kellomäki^2^, Edite Figueiras^6^, Jari Hyttinen^1^

^1^Computational Biophysics and Imaging Group, Faculty of Medicine and Health Technology, Tampere University, Arvo Ylpön katu 34, Tampere, Finland

^2^Biomaterials and Tissue Engineering Group, Faculty of Medicine and Health Technology, Tampere University, Tampere, Finland.

^3^Heart Group, Faculty of Medicine and Health Technology, Tampere University, Tampere, Finland

^4^Division of Pathology, Department of Laboratory Medicine, Karolinska Institutet, Stockholm, Sweden

^5^HAMK Smart Research Unit, Häme University of Applied Sciences, Hämeenlinna, Finland

^6^Champalimaud Research, Champalimaud Centre for the Unknown, Lisbon, Portugal

Corresponding author

Birhanu Belay

Arvo Ylpön katu 34, 33520, Tampere, Finland

[birhanu.belay@tuni.fi](about:blank),

+358402157322


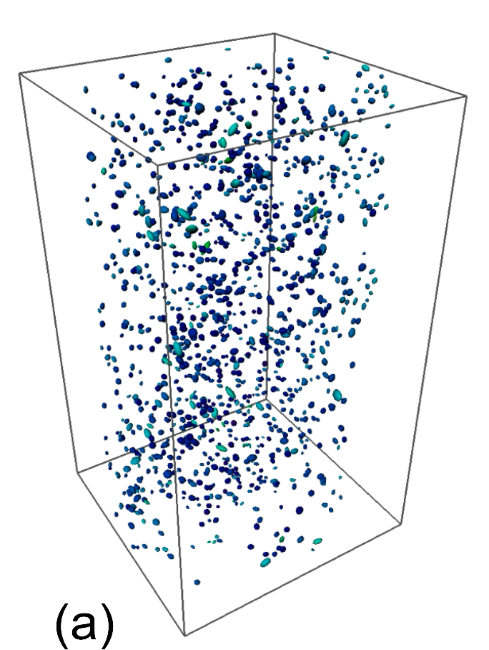

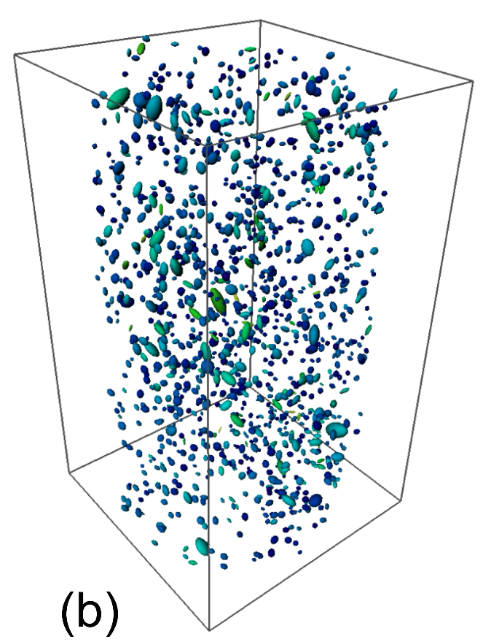
**
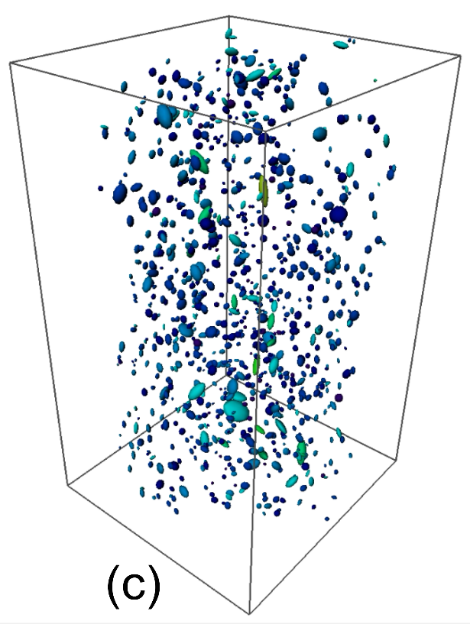
**

**Supplementary Video** **1.** The 3D reconstruction images of fibroblasts in (a) GG, (b) gelatin GG, and (c) Geltrex^®^ hydrogels.


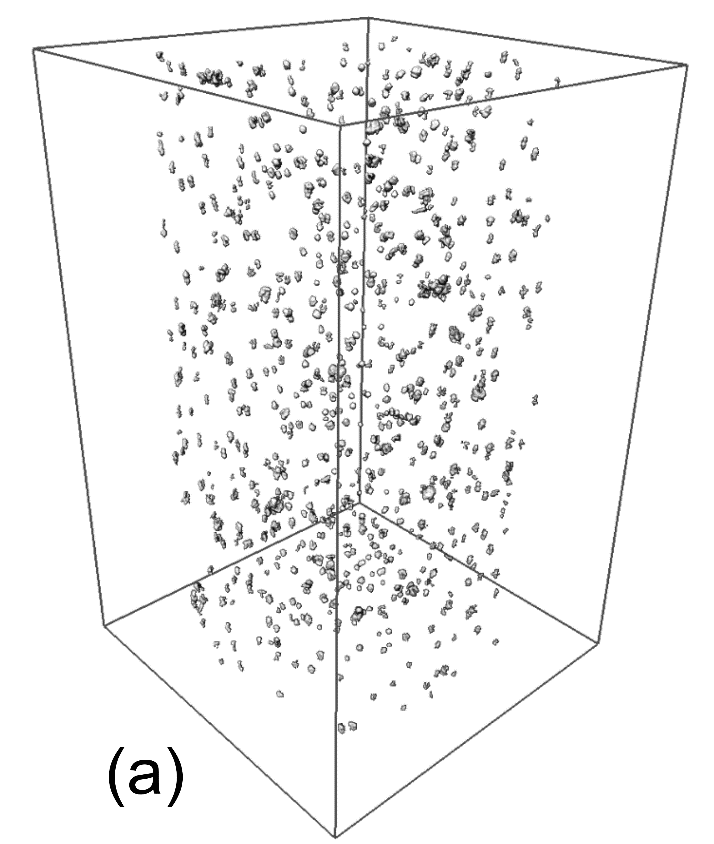

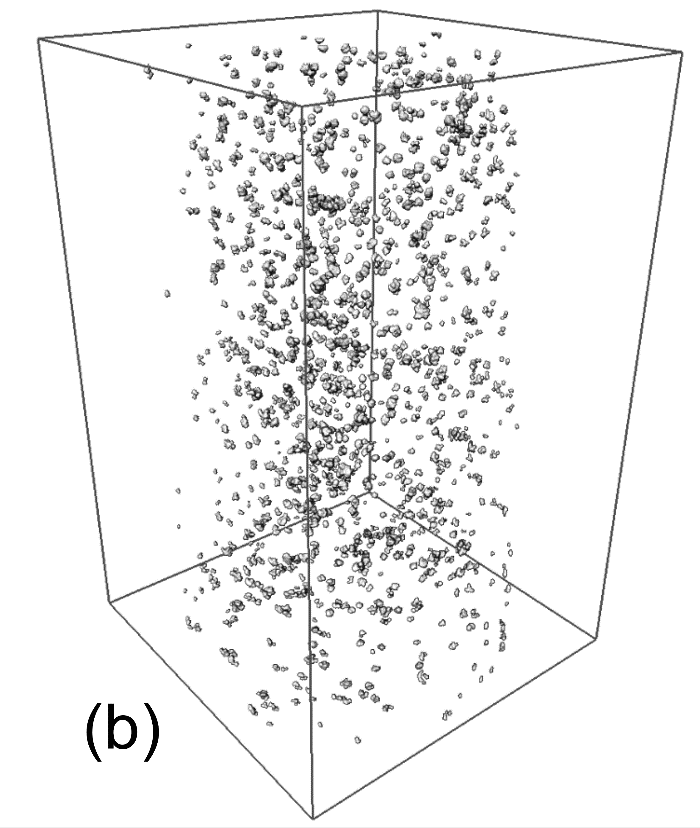
**
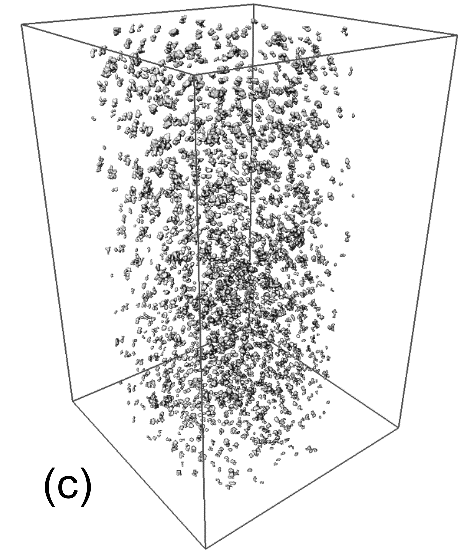
**

**Supplementary Video** **2.** The 3D reconstruction images of fibroblasts with a density of (a) 300 000 cells/mL, (b) 500 000 cells /mL, and 1000 000 cells/mL of GG hydrogel.


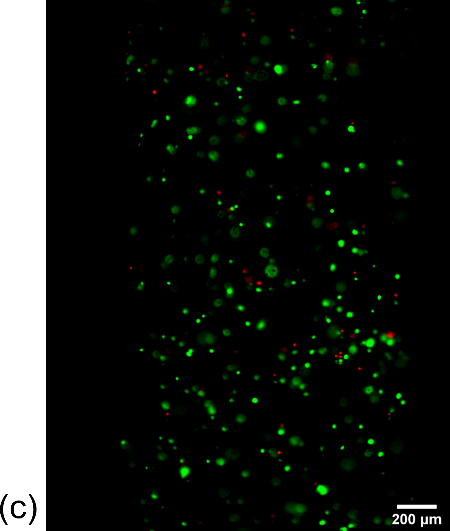

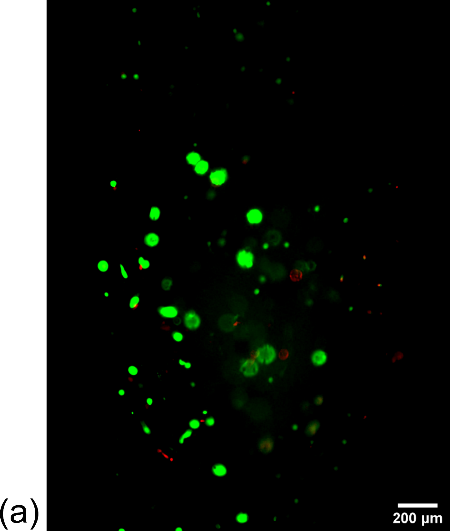

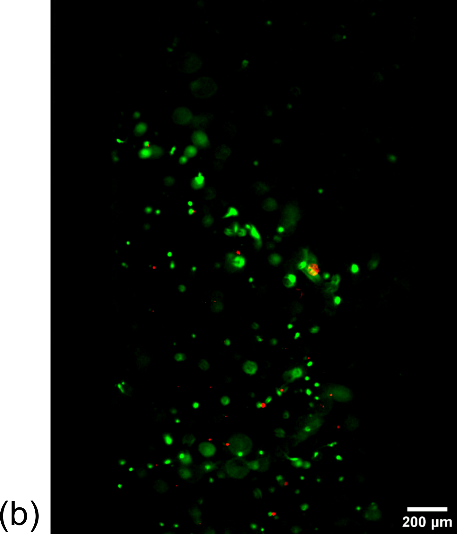


**Supplementary Video** **3.** Live/Dead fluorescence projection images of fibroblasts in (a) GG, (b) gelatin-GG, and (c) Geltrex^®^ hydrogels.


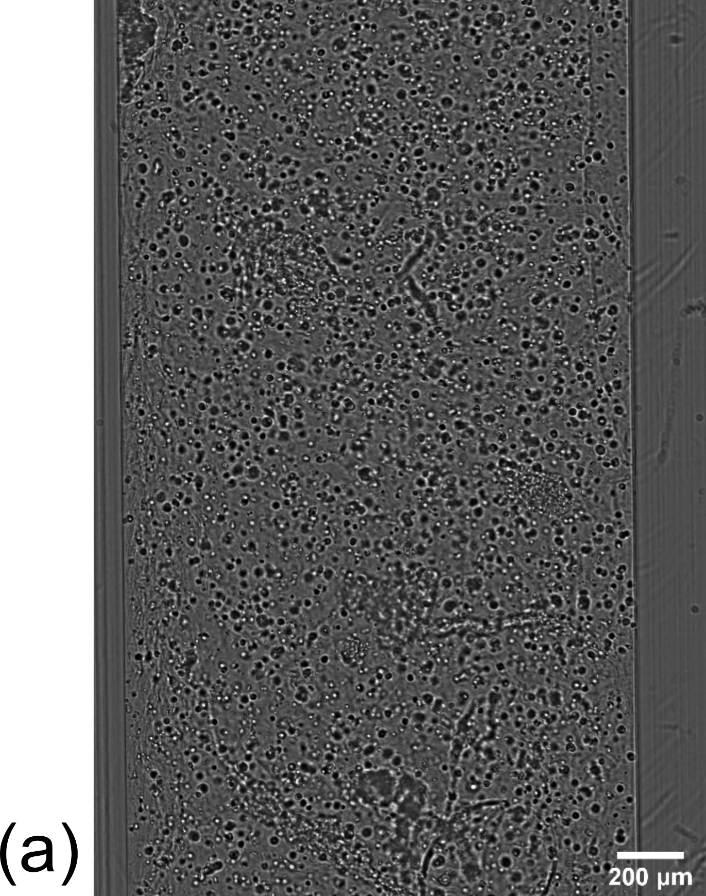

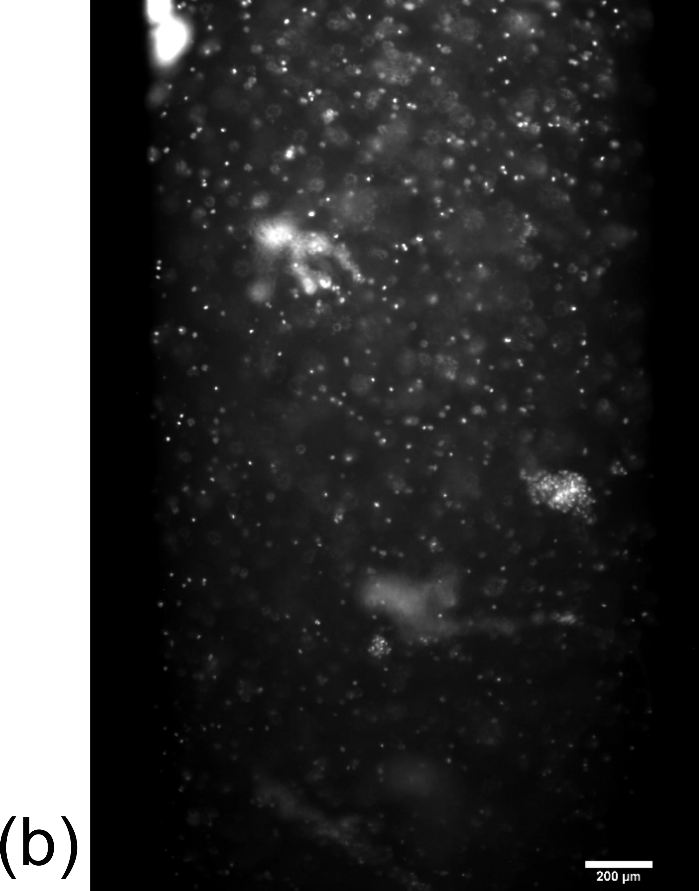


**Supplementary Video** **4.** OPT (a) bright field projection images of fibroblasts in GG and (b) fluorescence projection images of fibroblast nuclei.
